# Evaluation of the Influence of Protein Coatings on Bacterial Adhesion to Biomaterials

**DOI:** 10.1021/acs.jchemed.5c01262

**Published:** 2026-07-03

**Authors:** M. P. Ferraz, J. A. Loureiro

**Affiliations:** † Department of Mechanical Engineering, Faculty of Engineering, University of Porto, Rua Dr. Roberto Frias, 4200-465 Porto, Portugal; ‡ i3S - Institute for Research and Innovation in Health, University of Porto, Rua Alfredo Allen 208, 4200-135 Porto, Portugal; § INEB - Institute of Biomedical Engineering, University of Porto, Rua Alfredo Allen 208, 4200-135 Porto, Portugal; ∥ LEPABE - Laboratory for Process Engineering, Environment, Biotechnology and Energy, Faculty of Engineering, University of Porto, Rua Dr. Roberto Frias, 4200-465 Porto, Portugal; ⊥ ALiCE - Associate Laboratory in Chemical Engineering, Faculty of Engineering, University of Porto, Rua Dr. Roberto Frias, 4200-465 Porto, Portugal

**Keywords:** Graduate Education/Research, Interdisciplinary/Multidisciplinary, Laboratory Instruction, Physical Chemistry, Hands-On Learning/Manipulatives, Colloids, Nanotechnology, Lipids

## Abstract

Biomaterials used in medical implants are prone to bacterial adhesion and biofilm formation, which can lead to persistent infections. *Staphylococcus epidermidis*, which is a major cause of prosthesis-associated infections, adheres to biomaterial surfaces and forms biofilms, increasing resistance to antibiotics and immune responses, compromising the osseointegration. Protein adsorption on biomaterial surfaces plays a crucial role in bacterial adhesion. Serum albumin has the capacity to inhibit bacterial adhesion by creating a less favorable surface, whereas fibronectin (FN) promotes bacterial attachment by binding to bacterial fibronectin-binding proteins. This work studies the influence of human serum albumin (HSA) and FN coatings on adhesion of *S. epidermidis* to polycaprolactone (PCL) scaffolds, commonly used in tissue regeneration. Additionally, changes in surface hydrophilicity/hydrophobicity were analyzed. The findings provide the students with insight into how protein coatings modulate bacterial adhesion, potentially guiding the development of infection-resistant biomaterials, allowing them to gain a deeper understanding of biomaterials, strengthen their capacity for scientific creativity, and develop greater enthusiasm for research.

## Graphical abstract

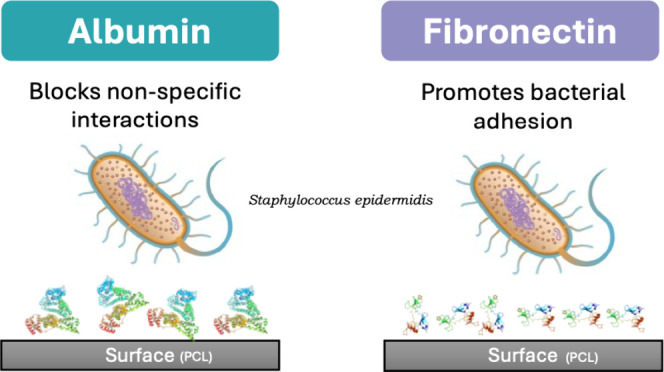

## Introduction

Biomaterials used to produce medical devices and implants are susceptible to bacterial adhesion, which can lead to persistent and difficult-to-treat infections. *Staphylococcus epidermidis*, a common skin commensal, is one of the primary responsible of device-associated infections due to its ability to adhere to and colonize biomaterial surfaces.^1,2^

The attachment of bacteria to human tissues or the surfaces of implanted biomaterials represents a critical step in the development of infections, allowing bacteria to multiply and colonize these surfaces.^3,4^ As bacterial microcolonies develop, certain strains, such as *S. epidermidis*, form biofilms after adhering to an implant. This layer turns the bacteria more resistant to the host’s immune system and reduces their susceptibility to antibiotics. The biofilm, which is composed primarily of exopolymers (mainly polysaccharides), may protect bacteria from antibiotic treatments, physiological forces, and possibly immune responses.^5^ So, these bacteria can persist on the material’s surface for extended periods without causing immediate issues. However, when conditions become favorable, such as a weakened immune response or insufficient tissue integration around the implant, the bacteria can multiply rapidly, leading to clinical infection.

Protein coating of biomaterials can significantly influence the adhesion and subsequent biofilm formation.^3,6^ Human serum albumin (HSA) is the most abundant protein in plasma, comprising about 60% of the total protein content. It consists of a single polypeptide chain with 585 amino acids, forming a largely helical structure composed of three major domains arranged in a heart-shaped configuration.^7^ Albumin performs several vital functions in the body, such as regulating osmotic pressure between blood and tissues, transporting various endogenous and exogenous molecules (e.g., fatty acids, hormones, calcium, drugs, and metabolites), and providing anti-inflammatory, antiapoptotic, antiplatelet, and antioxidant properties.^8^ Albumin coating onto material surfaces has demonstrated significant inhibition of bacterial adhesion to polymers, ceramics, and metals.^9^ The adsorption of serum albumin has been reported to reduce *S. epidermidis* adhesion by creating a less favorable surface for bacterial attachment.^10,11^ In contrast, human fibronectin (FN) is a large glycoprotein found in the extracellular matrix (ECM), made up of two nearly identical polypeptide chains, each with a molecular weight of around 250 kDa, connected by C-terminal disulfide bonds. FN is involved in several cellular processes, including cell adhesion, growth, differentiation, and migration, and plays a key role in cell attachment to biomaterial surfaces through its central RGD-binding sequence.^12^ FN has been shown to play a vital role in promoting bacterial adhesion to biomaterial surfaces. In recent decades, it has become evident that many bacteria have fibronectin-binding proteins (FnBPs), and these proteins are capable of attaching to an increasing number of sites within FN, which facilitates the initial attachment and subsequent biofilm development.^13–15^

Therefore, the purpose of this lab work was to investigate the influence of protein coating on a specific strain of *S. epidermidis’s* adherence to polycaprolactone (PCL) scaffolds, materials commonly used in tissue regeneration. Additionally, changes in hydrophilicity/hydrophobicity surface characteristics in the presence of these coatings were analyzed. The ultimate goal was to determine whether coating PCL surfaces with the two proteins, FN and HSA, affects the adherence of *S. epidermidis*.

This practical experiment is offered to third-year undergraduate bioengineering students at the Faculty of Engineering of the University of Porto (Portugal) during the Biological Systems Interfaces practical classes. This practical course is conducted in four 2 hour sessions, in which students follow all steps from sample preparation (Session 1) to bacterial adhesion quantification (Session 4) as presented in Table S2. A number of 16–18 students organized in groups of three or four elements attended each class.

## Experiment Overview

### Background

Medical implants have transformed the management of numerous pathologies, ranging from orthopedic reconstructions to cardiovascular and dental applications. Despite their clinical success, the long-term functionality of such devices is often threatened by infection risk, primarily due to bacterial colonization at the biomaterial interface. In particular, *S. epidermidis* is one of the leading causes of implant-associated infections.^16^ This opportunistic pathogen thrives on the surface of foreign materials, where it establishes biofilms that act as protective niches, increasing bacterial tolerance to antibiotics and shielding them from immune system clearance.^17^ Consequently, even localized colonization may evolve into persistent infections, frequently requiring implant removal and revision surgery.

The initial stages of bacterial adhesion are strongly dictated by the physicochemical properties of the biomaterial surface. When a material is introduced into the human body, rapid adsorption of proteins from blood and interstitial fluids occurs, forming a so-called “conditioning film”.^4,18^ This adsorbed protein layer is decisive in regulating microbial adhesion, as bacteria generally do not interact directly with the bare material but with the protein-coated surface. Depending on the identity and conformation of the adsorbed proteins, the surface can either become permissive or resistant to colonization. Among the proteins most relevant to this process, HSA and FN have been extensively investigated.

Another layer of complexity is introduced by the intrinsic material properties, particularly hydrophilicity and hydrophobicity. These characteristics influence both the orientation and the amount of protein adsorbed, and consequently, the extent of bacterial adhesion.^19^ Hydrophobic surfaces generally enhance nonspecific interactions with proteins, thereby facilitating microbial attachment. Hydrophilic surfaces, on the other hand, often yield thinner, less stable protein films that may reduce adhesion. Thus, by modulating wettability, it becomes possible to indirectly regulate bacterial colonization.

Polycaprolactone (PCL) is a semicrystalline, biodegradable polyester widely utilized in regenerative medicine and tissue engineering. Its mechanical flexibility, biocompatibility, and tunable degradation kinetics make it particularly attractive for scaffold fabrication.^20^ However, PCL is intrinsically hydrophobic, which may predispose it to unfavorable protein adsorption profiles and subsequent bacterial colonization. Surface modification strategies, such as protein precoating, have been proposed to mitigate these drawbacks. By selectively coating PCL scaffolds, it is possible to dissect how distinct protein layers modulate bacterial adhesion and biofilm development, and how these coatings alter the polymer’s surface wettability. Due to experimental limitations associated with the porous structure of PCL scaffolds, wettability characterization was performed on a model nonporous polymeric surface.

Understanding these interactions provides a mechanistic basis for designing infection-resistant biomaterials. By integrating knowledge of bacterial pathogenesis, protein adsorption dynamics, and biomaterial surface chemistry, researchers can develop implantable devices that not only support tissue regeneration but also minimize infection risk, ultimately enhancing the safety and longevity of medical implants.

### Reagents

Human serum albumin (HSA) (≥97%; 66.5 kDa), polycaprolactone (PCL) (80 kDa), 1,4-dioxane, and human plasma fibronectin (FN) (0.1% solution) were bought from Sigma-Aldrich (USA). Phosphate-buffered saline (PBS) (pH 7.4, 10 mM phosphate buffer, 2.7 mM potassium chloride, and 137 mM sodium chloride) was obtained from VWR International (USA). Tryptic soy broth (TSB), suitable for microbiological culture, was purchased from Difco (USA). Tryptic soy agar (TSa) was obtained from Merck Millipore (Germany). Brain heart infusion agar (BHI), suitable for bacteriological use, was bought from Liofilchem (Italy). All formulations were prepared using ultrapure water obtained from a Milli-Q system (Academic model, Millipore, France), and 70% ethanol was used for cleaning.

### Equipment

An orbital shaker oven (KS 4000 ic control, IKA), a UV–vis spectrophotometer (UV-1800, Shimadzu, Japan), an ultrasonic bath (Ultrasonic Cleaner VWR, 70 W, 45 kHz, VWR, USA), a centrifuge (Labofuge 400R, Heraeus, Germany), and an optical tensiometer (OCA 15, DataPhysics Instruments GmbH, Germany) were used.

### Experimental Details

The experimental plan was organized into four laboratory sessions, each lasting 120 minutes. The idea was to give students a step-by-step experience, starting from scaffold preparation and protein adsorption, moving to bacterial adhesion, and finishing with surface analysis and data treatment.

In the first session, students prepared the PCL scaffolds using the solvent-casting method. The second session was dedicated to bacterial adhesion experiments. The strain used was *S. epidermidis* RP62A, which was cultured and adjusted to the correct cell density. In the third session, attention shifted from microbiology to surface characterization. Students used a simple contact angle measurement to link protein adsorption on biomaterials to changes in hydrophobic or hydrophilic properties, which are known to influence bacterial adhesion. Finally, the fourth session was focused on data collection and analysis. After overnight incubation of the plated dilutions, colonies were counted, and the number of viable adherent bacteria was calculated. Each group worked with its own data set, but all results were shared among the class to create a larger pool of data for comparison. Students then applied basic statistical tools. All work was performed under close supervision to ensure proper handling of materials and the reproducibility of the experiments.

#### Preparation of Polycaprolactone Samples

PCL scaffolds were fabricated using the solvent-casting method. PCL granules (2% w/v) were dissolved in dioxane under constant stirring at room temperature (RT) until a homogeneous solution was obtained. The homogeneous PCL solution was then poured into a flat mold to achieve the desired scaffold geometry and rapidly frozen by placing samples in a −80 °C freezer for 12 h to ensure complete solidification of the PCL-solvent mixture. Frozen samples were transferred to a lyophilizer (freeze-dryer), where sublimation of the frozen dioxane was achieved under vacuum. Following lyophilization, the scaffolds were removed from the molds and immersed in deionized water to remove any residual solvent and enhance pore formation.

#### Protein Adsorption to PCL

PCL samples were cut into 3 mm cubes and washed twice with PBS at RT. PCL samples were placed in 96-well plates without treatment, with 100 μL of protein solution at 20 μg mL^–1^ for FN and 200 μg mL^–1^ for HAS in PBS, and incubated at 37 °C with gentle shaking for 60 min. The protein concentrations and incubation time were selected based on typical conditions reported in the literature to promote sufficient surface coverage.^21,22^ The 60 min incubation period was considered adequate to approach adsorption equilibrium for both proteins under the experimental conditions.

Three replicates were used for each experiment. Following incubation in the protein solution, each sample was gently washed twice with PBS at RT to remove nonadsorbed or loosely adsorbed protein.

#### Bacterial Adhesion to the PCL Samples

*S. epidermidis*, reference strain RP62A (ATCC 35984), known for producing biofilm, was the chosen strain for the lab work. The bacterial strain was stored at −70 °C in a cryopreservation solution composed of 70% TSB and 30% glycerol (diluted 1:1 with water). For cultivation, *S. epidermidis* strain was cultured on BHI agar at 37 °C for 24 h. All media were prepared following the manufacturer’s instructions.

To study bacterial adhesion, bacteria were first grown in 15 mL TSB and incubated at 37 °C for 24 h. After incubation, 50 μL of the bacterial culture was transferred to a tube containing 30 mL of fresh TSB and incubated again at 37 °C with gentle shaking for 18 h to achieve a stationary phase. Bacterial cells were then collected by centrifugation for 5 min at 10 N and assigned to each group. The harvested cells were washed twice and resuspended in a saline solution (0.9% NaCl in distilled water) at a final concentration of 1.0 × 10^8^ colony-forming units (CFU)/mL, following the McFarland standard (BioMerieux, France).

PCL samples, either uncoated or coated with protein, were placed into 96-well plates with the bacterial suspension and incubated at 37 °C with gentle shaking for 60 min. Each experiment was performed in triplicate. After incubation, the samples were gently rinsed twice with PBS to remove any nonadherent or loosely attached bacteria.

#### Colony-Forming Unit Counting

After washing, the samples were placed in clean wells with 100 μL of sterile PBS. The plates were subjected to sonication for 30 s in an ultrasonic water bath. This process helped to detach the adhered bacterial cells. Serial dilutions (10×, 100×, and 1000×) of the sonicated solutions were then prepared in PBS and plated on TSA. After 24 h of incubation at 37 °C, the number of bacterial colonies that adhered to the material was counted. CFU counts were considered between 10 and 100. As a control, the material samples were also plated after sonication to confirm that all adhering bacteria had been successfully removed by the procedure.

Each group quantified the number of CFUs based on the different successive dilutions. The results were shared among the groups so that everyone had access to all the groups’ results.

#### Contact Angle Measurements

Due to the highly porous and rough structure of the PCL scaffolds obtained by solvent casting and lyophilization, reliable contact angle measurements could not be performed, as liquid droplets tend to penetrate into the porous network rather than forming a stable interface. To overcome this limitation, high-density polyethylene (HDPE), a nonporous and smooth polymeric substrate, was used as a model surface for wettability characterization.

Importantly, the aim of these measurements was to assess the relative effect of protein adsorption (HSA and FN) on surface hydrophilicity/hydrophobicity under controlled conditions, rather than to determine the absolute wettability of the PCL scaffolds.

The HDPE samples undergo the same procedure as the PCL. Briefly, the HDEPE samples were immersed for 60 min at 37 °C in solutions of HSA at 200 μg/mL or FN at 20 μg/mL, followed by washing three times with PBS. Then the contact angle was recorded for the surface-treated HDPE and untreated HDPE (used as a control).

Contact angle measurements were carried out using an optical tensiometer. The surface tension of PBS was first determined by the pendant drop method. After this step, 5 μL PBS droplets were deposited on the HDPE previously treated with HSA or FN. Each measurement was repeated three times on different regions of the same sample to account for surface heterogeneity.

Once the liquid surface tension and the contact angle on a given solid surface are known, it is possible to estimate the work of adhesion (*W*_a_) at the solid–liquid interface (γ_LG_) using the Young–Dupré equation (eq 1):^23^

1 $$W_{a}=\gamma_{\mathrm{LG}}\left(1+\cos\theta \right)$$

Additionally, the work of cohesion (*W*_c_), which represents the intermolecular forces within the liquid phase, can be determined according to eq 2:

2 $$W_{c}=2\gamma_{\mathrm{LG}}$$

#### Statistical Analysis

All groups performed the statistical analysis of the counted CFU results. Student’s *t* tests with a 95% confidence interval were employed to assess whether there were significant differences between groups; *p* values below 0.05 were used to identify significant differences.

## Hazards

Most of the work in this study involves standard lab techniques that are generally safe. The one area that requires extra care is making PCL scaffolds using the solvent-casting method. 1,4-Dioxane is a flammable solvent that can be dangerous if it is inhaled or touches the skin (https://www.sigmaaldrich.com/PT/en/product/mm/589591). To stay safe, all handling of this chemical should happen inside a certified fume hood. Appropriate personal protective equipment (PPE), including a lab coat, nitrile gloves, and safety glasses, should be used. Any leftover solvents should go into labeled containers and be disposed of according to the lab’s hazardous waste rules.

Working with proteins like HSA and FN is low-risk. The experiments with *S. epidermidis* are the most sensitive. Even though the ATCC 35984 strain is well-known, it is classified as biosafety level 2 because it can form biofilms and may cause infections in certain conditions. All bacterial handling should be done using aseptic techniques. Ideally, work is performed inside a Class II biological safety cabinet, and PPE is mandatory. Surfaces should be cleaned before and after experiments with a disinfectant like 70% ethanol. Extra caution is needed when using sonication or working with bacterial suspensions to prevent aerosols. Plates or containers should stay covered whenever possible. Follow all instructions for ultrasonic water baths carefully. All biological waste, including pipet tips, agar plates, and bacterial cultures, must be autoclaved before disposal. Hands should be washed thoroughly after handling any bacteria.

## Results and Discussion

### Bacterial Adhesion Evaluation

This study aimed to examine the influence of albumin and fibronectin coating on the initial adhesion of *S. epidermidis* to PCL. Albumin and FN were examined in this study due to their high abundance in plasma and their contrasting effects on bacterial attachment to biomaterial surfaces. As a first step, the PCL structures were in contact with the albumin and FN. After that, three dilutions were performed, and the CFU were counted in the dilutions having between 10 and 100 CFU. A comparison between HSA and FN coating on PCL regarding bacterial adhesion was assessed and is presented in Figure 1.

**Figure 1:**
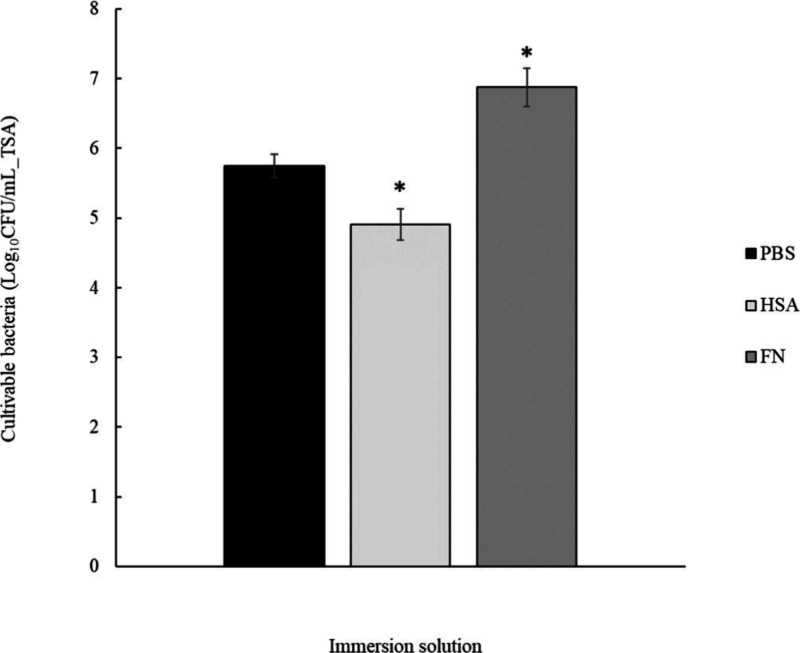
Results presented as the average of four work groups and expressed as log_10_(CFU/mL) ± SD. * indicates a statistical difference compared to the noncoated PCL samples (*p* < 0.05) (*n* = 4).

The presence of HSA coating on PCL led to a reduction of *S. epidermidis* adhesion from log_10_(CFU/mL) = 5.74 ± 0.17 to 4.91 ± 0.22. The reduction of bacterial adhesion in materials previously coated with HSA aligns with findings from previously reported studies, suggesting that albumin forms a protein layer that limits bacterial access to the underlying surface.^6^ This antiadhesive effect is thought to result from steric hindrance and the modulation of surface charge, which collectively create an environment less favorable for bacterial colonization.^24^ Understanding these interactions is crucial for improving the design of biomaterials intended for medical applications, where preventing bacterial attachment is a key strategy to reduce the risk of device-associated infections.

In contrast, FN coating on PCL resulted in an increase in bacterial adhesion (log_10_(CFU/mL) = 6.88 ± 0.27) compared to PCL without the protein (log_10_(CFU/mL) = 4.91 ± 0.22). The adhesion of *S. epidermidis* in the presence of FN-coated polymer surfaces increases bacterial adhesion, also aligning with previous studies.^25^ In fact, FN is a highly abundant plasma and extracellular matrix protein that provides specific binding sites recognized by bacterial surface proteins, facilitating its attachment. The presence of a FN layer on biomaterials can act as a bridge between the bacteria and the underlying surface, promoting colonization and potentially contributing to early biofilm formation.

This effect highlights the dual role of plasma proteins in modulating bacterial interactions with biomaterials, where some proteins, like albumin, can hinder adhesion, while others, such as FN, may actively improve it. Understanding these dynamics is essential for designing biomaterial surfaces that minimize the risk of device-associated infections.

### Effect of Surface Coating with Proteins on the Material’s Hydrophobicity/Hydrophilicity

When a liquid droplet is deposited onto a solid surface, two main outcomes may occur: either the liquid spreads completely across the surface or a finite angle is formed between the solid surface and the tangent to the liquid droplet. This angle is defined as the contact angle (θ) where γ_LG_ represents the surface tension in the liquid–gas interface (Figure 2). In this case, a three-phase contact line is established at the solid–liquid–gas interface, also referred to as the wettability line.^26^ The contact angle can vary between two theoretical limits. A contact angle of 0° corresponds to complete wetting of the surface, where the liquid spreads entirely, indicating a highly hydrophilic surface. In contrast, a contact angle of 180° represents complete nonwetting, where the liquid forms a nearly spherical droplet, indicating a highly hydrophobic surface.

**Figure 2:**
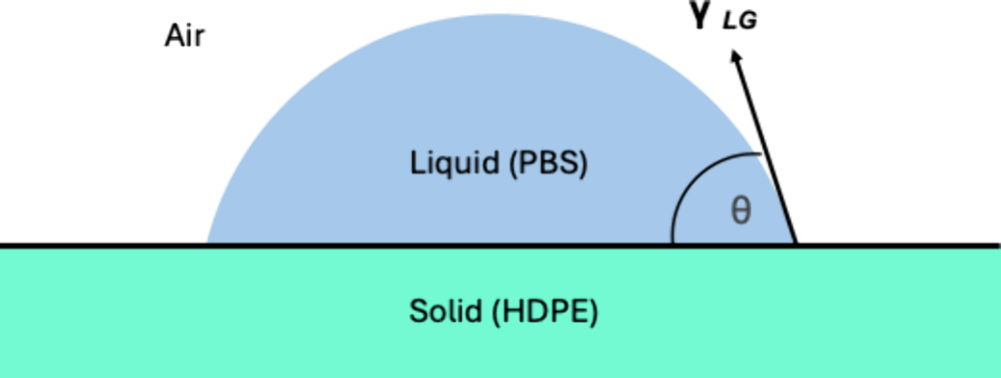
Profile of a liquid droplet forming a contact angle θ on a solid surface.

It should be noted that contact angle measurements were performed on HDPE substrates rather than directly on PCL scaffolds. This approach was adopted due to the highly porous morphology of PCL, which prevents accurate wettability measurements. Therefore, HDPE was used as a model flat surface to evaluate the effect of protein adsorption on surface wettability. While absolute values may differ between materials, the trends observed upon protein adsorption are expected to be representative of general polymer–protein interactions.

The surface tension obtained for the PBS was 72.33 ± 0.42 mN/m. The contact angles were measured for the polymer HDPE coated with FN and HSA (Table 1). The contact angle measurements between the HDPE surfaces and PBS buffer clearly demonstrate that the protein coatings affect the surface hydrophobicity.

**Table 1:** Contact Angles Obtained between the Liquid PBS and the Uncoated HDPE, HSA-Coated HDPE, and FN-Coated HDPE at Room Temperature (∼21°C) (*n* = 3)

Coating	Contact Angle (deg)
uncoated	89.96 ± 5.56
HSA	45.03 ± 1.72
FN	51.33 ± 0.93

The unmodified HDPE surface exhibited a contact angle of 89.96 ± 5.56°, consistent with the known behavior of HDPE. This polymer is composed mainly of nonpolar methylene groups (−CH_2_−), which confer low affinity for polar molecules such as water. The relatively high contact angle reflects the hydrophobic character of the uncoated HDPE, as polar liquids tend to minimize interactions with apolar surfaces, resulting in higher interfacial tension.^27^

Coating the HDPE with proteins led to a significant decrease in the contact angles, indicating increased surface hydrophilicity. HSA is a water-soluble globular protein containing numerous polar and charged groups, such as amino and carboxyl groups.^27^ When adsorbed onto the HDPE surface, HSA likely orients itself in a manner that exposes these polar residues to the aqueous environment, enhancing interactions with water molecules in PBS. This is consistent with the observed contact angle of 45.03 ± 1.72°, showing a substantial increase in hydrophilicity compared to the uncoated HDPE. Such behavior aligns with previous studies reporting that protein adsorption can significantly alter surface energy and reduce the intrinsic hydrophobicity of polymeric substrates.^28,29^

The HDPE surface coated with FN displayed a contact angle of 51.33 ± 0.93°, slightly higher than that of the HSA-coated surface but considerably lower than the uncoated HDPE. FN contains both polar regions that interact with water and hydrophobic regions that may interact strongly with the polymer substrate. Its adsorption may lead to partial exposure of hydrophobic domains, limiting the accessibility of polar residues to the aqueous phase. This explains the intermediate contact angle, suggesting moderate hydrophilicity. These results highlight that the chemical composition and spatial arrangement of polar and nonpolar domains in the protein are key factors in determining surface energy and hydrophilic behavior.

Also, the work of adhesion and cohesion was determined (Table 2). The calculated thermodynamic parameters provide additional insight into the interfacial behavior of the different surfaces. The uncoated HDPE showed the lowest work of adhesion (72.38 ± 7.01 mJ/m^2^), consistent with its hydrophobic and apolar nature, whereas protein adsorption markedly increased work of adhesion to 123.44 ± 1.53 mJ/m^2^ for HSA and 117.52 ± 0.91 mJ/m^2^ for FN. These higher values confirm that both proteins enhance the affinity of the surface for the aqueous phase, in agreement with the lower contact angles measured. The work of cohesion was 144.67 ± 0.83 mJ/m for all conditions since just depends of the surface tension of the liquid. This value reflects the intrinsic cohesive forces within the liquid phase. The fact that adhesion work values for protein-coated surfaces approached cohesion work highlights the strong wetting tendency of these modified interfaces.

**Table 2:** Work of Cohesion (*W*_c_) and Adhesion (*W*_a_) between the Liquid PBS and the Uncoated HDPE, HSA-Coated HDPE, and FN-Coated HDPE at Room Temperature (∼21°C) (*n* = 3)

Coating	*W*_a_ (mJ/m^2^)	*W*_c_ (mJ/m^2^)
uncoated	72.38 ± 7.01	144.67 ± 0.83
HSA	123.44 ± 1.53	
FN	117.52 ± 0.91	

So, even our contact angle data showed that both HSA and FN coatings significantly increased the hydrophilicity of HDPE compared to the untreated polymer, yet these coatings had opposite effects on bacterial adhesion, since *S. epidermidis* showed an increase in adhesion to FN-coated polymer, whereas HSA-coating inhibited bacterial attachment.

This apparent discrepancy between surface wettability and adhesion can be explained by the dual role of the conditioning protein layer. On the one hand, changes in hydrophobicity influence nonspecific interactions between bacterial cell walls and the material surface. The unmodified PCL and HDPE are highly hydrophobic, and such surfaces are generally favorable for nonspecific bacterial attachment. Both HSA and FN coatings reduced the contact angle, indicating higher hydrophilicity, which by itself would normally reduce adhesion through the formation of a hydrated interfacial layer that limits van der Waals and hydrophobic interactions. However, the biochemical identity of the adsorbed protein proved to be more important than wettability alone.

Overall, the work of adhesion and cohesion measurements confirms that protein adsorption substantially alters the interfacial properties of HDPE. The increase in Wa observed for both HSA and FN underscores their role in enhancing the thermodynamic driving force for wetting and interfacial stability. However, the biological consequences diverge: while HSA increases Wa and creates a hydrated, nonadhesive interface, fibronectin combines enhanced interfacial energy with the presentation of bacterial binding sites, thereby facilitating colonization.

These findings highlight the importance of considering both surface energetics and molecular specificity when evaluating the biological performance of protein-coated biomaterials (Table 3).

**Table 3:** Main Advantages and Disadvantages of Using HSA Coatings versus FN Coatings

HSA	FN
**Interaction with *S. epidermidis***	
Create a passivating surface, limiting bacterial adhesion	Enhances bacterial adhesion due to specific interactions with bacterial adhesions
Acts as a nonspecific protein layer, reducing likelihood of *S. epidermidis* attachment	Can promote bacterial colonization on biomaterial surfaces
**Advantages**	
Reduces the risk of *S. epidermidis* biofilm formation	Could support applications requiring specific cellular adhesion in bacterial contamination is controlled
**Disadvantages**	
Less effective in facilitating cellular integration, focusing solely on bacterial inhibition	Increases the risk of *S. epidermidis* biofilm formation and implant-related infections

## Pedagogical Assessment

The practical teaching course in Biological Systems Interfaces was offered to classes of 16–18 students, typically divided into working groups, each composed of three or four students. In our practical examples, we present a work schedule and results from a class of 16 students divided into four groups. Before starting the experimental work, students were asked to review the Introduction and Background section of the Laboratory Guide, which provided the theoretical framework for surface modification and protein adsorption. During this preparatory phase, the instructor addressed questions and clarified key concepts regarding hydrophobicity, wettability, and bacterial adhesion mechanisms.

Over the course of four laboratory sessions, students were introduced to the methodologies for preparing protein-coated polymer surfaces. They performed experiments for the assessment of bacterial adhesion using *S. epidermidis*. They also conducted contact angle measurements to evaluate surface wettability and calculated the corresponding adhesion and cohesion work, gaining practical experience in interpreting thermodynamic parameters of solid–liquid interactions. So, the whole experience allows students to establish correlations between microbial colonization, protein adsorption, and surface energy.

After completing the laboratory tasks, students were invited to present their experimental data and engage in critical analysis of the results, particularly focusing on the contrasting effects of HSA and FN. These discussions encouraged students to reflect on both the physicochemical and biological dimensions of biomaterial surfaces.

All procedures were supervised by the instructor, who provided guidance and feedback during each experimental step. Student performance was evaluated across several competencies, including technical execution, data analysis, critical thinking, teamwork, and adherence to laboratory safety guidelines.

At the end of the set of the laboratory classes, students were expected to have acquired the following specific competencies:

- Prepare surfaces coated with proteins
- Assess bacterial adhesion on different biomaterial surfaces
- Perform and interpret contact angle measurements
- Calculate and analyze the work of adhesion and cohesion
- Correlate biological outcomes with physicochemical surface properties
- Apply critical thinking to interpret discrepancies between theory and experiment
- Develop collaborative and problem-solving skills in a laboratory environment

To evaluate changes in student learning outcomes, students completed a pretest before the laboratory activity and a post-test at the end of the laboratory module. The pretest consisted of the same true/false conceptual questions included in Group I of the final assessment, allowing a direct comparison between students’ baseline understanding and their performance after completing the laboratory activity. These questions addressed key concepts related to biomaterial-associated infections, bacterial adhesion quantification, protein coatings, contact angle interpretation, and adhesion/cohesion work.

The post-test also included additional conceptual and data-interpretation questions directly aligned with the pedagogical objectives of the laboratory activity. Specifically, students were evaluated on their ability to do the following:

(i) Understand the role of protein adsorption in modulating bacterial adhesion
(ii) Calculate and analyze adhesion and cohesion work
(iii) Interpret contact angle measurements and relate them to surface wettability
(iv) Critically relate physicochemical properties with biological outcomes

Comparison of the pre- and post-test responses showed an increase in the percentage of correct answers in Group I, from 54.6 ± 13.9% in the pretest to 85.6 ± 14.4% in the post-test (*n* = 57), indicating gains in students’ conceptual understanding after participation in the laboratory activity. Improvements were particularly evident for questions related to bacterial adhesion quantification, the role of protein coatings, and interpretation of wettability-related parameters. These results provide direct evidence that participation in the laboratory activity contributed to measurable changes in student learning outcomes. Students’ performance in the post-test was evaluated based on the correctness of calculations, clarity of reasoning, and ability to interpret experimental results (see Supporting Information (SI) sections 8 and 9). Overall, most students demonstrated a good level of understanding, with the majority achieving correct or partially correct responses across the different question types. In particular, students showed strong performance in quantitative calculations and in identifying the contrasting roles of HSA and fibronectin in bacterial adhesion, while some difficulties were observed in more advanced conceptual integration tasks (see SI section 10).

These results indicate that the laboratory activity was effective in achieving its intended pedagogical goals, particularly in promoting the integration of physicochemical and biological concepts.

## Conclusion

Graduate students from the bachelor’s in bioengineering had the opportunity to carry out a laboratory project focused on the modification of polymeric biomaterials. Students also assessed the adhesion of *S. epidermidis* to PCL surface-modified with different proteins, the HSA and FN. To link biological performance with physicochemical properties, students applied a simplified protocol to evaluate surface wettability. However, the results demonstrate that hydrophilicity alone does not determine bacterial adhesion. Although both HSA and FN coatings increased surface hydrophilicity, they led to opposite effects on S. epidermidis adhesion.

This highlights that bacterial adhesion is governed not only by physicochemical properties such as wettability, but also by specific protein–bacteria interactions.By comparing HSA- and FN-coatings, students understood the dual role of protein layers. Taken together, the obtained results highlight that surface hydrophilicity, although an important determinant of bacterial adhesion, cannot be interpreted in isolation. The type of protein adsorbed onto the biomaterial critically defines the biological outcome: while albumin acts as a protective, nonadhesive coating, fibronectin functions as a biological ligand that promotes colonization by *S. epidermidis*. This distinction underscores the relevance of protein-specific interactions in the early stages of biofilm formation and helps to explain why biomaterials exposed to blood or extracellular matrix proteins may exhibit very different susceptibilities to bacterial colonization, depending on which proteins dominate the conditioning layer.

Finally, this project highlighted the relevance of surface engineering in the field of biomaterials and medical devices. Students gained hands-on experience in correlating surface energy, wettability, and bacterial adhesion, which are fundamental aspects for the design of anti-infective biomaterials. The activity provided them with a broader understanding of how physicochemical and biological parameters are interconnected, preparing them to further explore applications of biomaterials in therapeutic and biomedical contexts.

## Supplementary Material





## Notes

Objectives of the laboratory classes, background, recommended literature, laboratory safety procedures, reagents, planning of the sessions, experimental procedure, results and discussion, answers to questions, students’ grades, and notes for instructors (PDF, DOCX)

## References

[1] Barros J., Grenho L., Manuel C. M., Ferreira C., Melo L., Nunes O. C., Monteiro F. J., Ferraz M. P. (2014). Influence of nanohydroxyapatite
surface properties on *Staphylococcus epidermidis* biofilm formation. J. Biomater. Appl..

[2] Cheung G. Y. C., Otto M. (2025). *Staphylococcus epidermidis*-key to understanding biofilms, commensalism, and more. J. Bacteriol..

[3] Barros J., Monteiro F. J., Ferraz M. P. (2022). Bioengineering Approaches to Fight
against Orthopedic Biomaterials Related-Infections. Int. J. Mol. Sci..

[4] Kreve S., Reis A. C. D. (2021). Bacterial adhesion to biomaterials: What regulates
this attachment? A review. Jpn. Dent. Sci. Rev..

[5] Le K. Y., Park M. D., Otto M. (2018). Immune Evasion
Mechanisms of *Staphylococcus epidermidis* Biofilm Infection. Front. Microbiol..

[6] Ribeiro M., Monteiro F. J., Ferraz M. P. (2012). Staphylococcus
aureus and *Staphylococcus epidermidis* adhesion to nanohydroxyapatite
in the presence of model proteins. Biomed. Mater..

[7] Mishra V., Heath R. J. (2021). Structural and Biochemical
Features of Human Serum
Albumin Essential for Eukaryotic Cell Culture. Int. J. Mol. Sci..

[8] Belinskaia D. A., Voronina P. A., Goncharov N. V. (2021). Integrative Role of Albumin: Evolutionary,
Biochemical and Pathophysiological Aspects. J. Evol. Biochem. Physiol..

[9] Chen X., Zhou J., Qian Y., Zhao L. (2023). Antibacterial coatings
on orthopedic implants. Mater. Today Bio.

[10] Martin M. L., Pfaffen V., Valenti L. E., Giacomelli C. E. (2018). Albumin
biofunctionalization to minimize the Staphylococcus aureus adhesion
on solid substrates. Colloids Surf., B.

[11] Ardehali R., Shi L., Janatova J., Mohammad S. F., Burns G. L. (2003). The inhibitory activity
of serum to prevent bacterial adhesion is mainly due to apo-transferrin. J. Biomed. Mater. Res., Part A.

[12] Dalton C. J., Lemmon C. A. (2021). Fibronectin: Molecular
Structure, Fibrillar Structure
and Mechanochemical Signaling. Cells.

[13] Speziale P., Arciola C. R., Pietrocola G. (2019). Fibronectin
and Its Role in Human
Infective Diseases. Cells.

[14] Joh D., Wann E. R., Kreikemeyer B., Speziale P., Hook M. (1999). Role of fibronectin-binding
MSCRAMMs in bacterial adherence and entry into mammalian cells. Matrix Biol..

[15] Hook M., McGavin M. J., Switalski L. M., Raja R., Raucci G., Lindgren P. E., Lindberg M., Signas C. (1990). Interactions of bacteria
with extracellular matrix proteins. Cell Differ.
Dev..

[16] Severn M. M., Horswill A. R. (2023). *Staphylococcus epidermidis* and its dual lifestyle in skin health and infection. Nat. Rev. Microbiol..

[17] Oliveira A. S., Saraiva L. M., Carvalho S. M. (2023). *Staphylococcus epidermidis* biofilms undergo metabolic
and matrix remodeling under nitrosative
stress. Front. Cell. Infect. Microbiol..

[18] Bhagwat G., O’Connor W., Grainge I., Palanisami T. (2021). Understanding
the Fundamental Basis for Biofilm Formation on Plastic Surfaces: Role
of Conditioning Films. Front. Microbiol..

[19] Yuan Y., Hays M. P., Hardwidge P. R., Kim J. (2017). Surface characteristics
influencing bacterial adhesion to polymeric substrates. RSC Adv..

[20] Ntrivala M. A., Pitsavas A. C., Lazaridou K., Baziakou Z., Karavasili D., Papadimitriou M., Ntagkopoulou C., Balla E., Bikiaris D. N. (2025). Polycaprolactone
(PCL): the biodegradable polyester shaping the future of materials
– a review on synthesis, properties, biodegradation, applications
and future perspectives. Eur. Polym. J..

[21] Barberi J., Mandrile L., Napione L., Giovannozzi A. M., Rossi A. M., Vitale A., Yamaguchi S., Spriano S. (2022). Albumin and fibronectin adsorption on treated titanium
surfaces for osseointegration: An advanced investigation. Appl. Surf. Sci..

[22] Li Y., Ji R., Li Y., Li J., Chen H. (2024). Early-Stage Protein
Adsorption Sequence on Blood-Contacting Surfaces: Answer to Vroman’s
Question. Anal. Chem..

[23] Erbil, H. Y. Surface Chemistry of Solid and Liquid Interfaces; Blackwell, 2006.

[24] Xu X., Hu J., Xue H., Hu Y., Liu Y.-n., Lin G., Liu L., Xu R.-a. (2023). Applications
of human and bovine serum albumins in
biomedical engineering: A review. Int. J. Biol.
Macromol..

[25] Xu L.-C., Siedlecki C. A. (2012). Effects
of Plasma Proteins on *Staphylococcus
epidermidis* RP62A Adhesion and Interaction with Platelets
on Polyurethane Biomaterial Surfaces. J. Biomater.
Nanobiotechnol..

[26] Extrand C. W. (2017). Comment
on Solid–Liquid Work of Adhesion. Langmuir.

[27] Xu Y., Luo Z., Tao Y., Xu M., Liao J. (2022). Transforming hydrophobicity
of high-density polyethylene surface to hydrophilicity and superoleophobicity
by surface grafted with polyvinyl alcohols for oil contaminants cleanup. Colloids Surf., A.

[28] Stallard C. P., McDonnell K. A., Onayemi O. D., O’Gara J. P., Dowling D. P. (2012). Evaluation of protein adsorption on atmospheric plasma
deposited coatings exhibiting superhydrophilic to superhydrophobic
properties. Biointerphases.

[29] Jonas S. J., Stieg A. Z., Richardson W., Guo S., Powers D. N., Wohlschlegel J., Dunn B. (2015). Protein Adsorption
Alters Hydrophobic
Surfaces Used for Suspension Culture of Pluripotent Stem Cells. J. Phys. Chem. Lett..

