# Supplementary material for: Evaluation of the Influence of Protein Coatings on Bacterial Adhesion to Biomaterials
Source: J Chem Educ. 2026 Jul 3;103(8):4448–55. doi: 10.1021/acs.jchemed.5c01262 (PMC13480223; doi:10.1021/acs.jchemed.5c01262)
Supplement: Supplementary file 1 [file ed5c01262_si_001.pdf]

Supporting Information

Evaluation of the influence of protein coatings on bacterial adhesion to biomaterials

Ferraz, M.P.[a, b, c] and Loureiro, J.A.[a, d, e*]

[a] Department of Mechanical Engineering, Faculty of Engineering, University of Porto, Rua Dr. Roberto Frias, 4200-465 Porto, Portugal

[b] i3S - Institute for Research and Innovation in Health, University of Porto, Rua Alfredo Allen, 208, 4200-135, Porto, Portugal

[c] INEB - Institute of Biomedical Engineering, University of Porto, Rua Alfredo Allen, 208, 4200-135, Porto, Portugal

[d] LEPABE - Laboratory for Process Engineering, Environment, Biotechnology and Energy, Faculty of Engineering, University of Porto, Rua Dr. Roberto Frias, 4200-465 Porto, Portugal

[e] ALiCE - Associate Laboratory in Chemical Engineering, Faculty of Engineering, University of Porto, Rua Dr. Roberto Frias, 4200-465 Porto, Portugal

*Corresponding author: jasl@fe.up.pt

Table of Contents

1. Objectives of the Laboratory Classes 2
2. Background 3
3. Recommended Literature 4
4. Laboratory Safety Procedures 5
5. Reagents...5
6. Planning of the Sessions 6
7. Experimental Procedure 7
8. Results Discussion 11
9. Answers to Questions 15
10. Students' Grades 19
11. Notes for the Instructors 21

1. Objectives of the Laboratory Classes:

- Preparation of a biomaterial coated with proteins.
- Determination of surface tension of a buffer that simulates biological fluids (PBS) by the suspended drop method.
- Determination of contact angle values on a solid surface.
- Interpretation of contact angle values obtained based on the protein coat applied to the surface.
- Familiarization with working in aseptic conditions.
- Quantification of the cells adhered to the biomaterials.
- Evaluation of the significance of the obtained results.

2. **Background**:

Implantable biomaterials play an essential role in regenerative medicine and tissue engineering, enabling the repair and replacement of biological tissues. Among the polymers used, polycaprolactone (PCL) stands out due to its biocompatibility, biodegradability, and ease of processing.

Different surface modification methods are applied to optimize the interaction between PCL and biological tissues. Techniques such as plasma, chemical treatments, polymer grafting, and functionalization with biomolecules are explored to improve cell adhesion and modulate the biological response to the implant.

A crucial step after the implantation of a biomaterial is the adsorption of proteins on the surface. The composition and organization of these proteins directly influence cellular processes, such as adhesion, proliferation, and differentiation. However, protein adhesion also affects bacterial colonization and may favor or inhibit the formation of biofilms and infections associated with implants.

Understanding these interactions between biomaterials, proteins, and bacteria is essential for the development of strategies that reduce infectious complications and improve the integration of implants into the body.

In this laboratory class, an implantable material will be coated with a protein, and the bacterial adhesion will be evaluated (Figure S1).

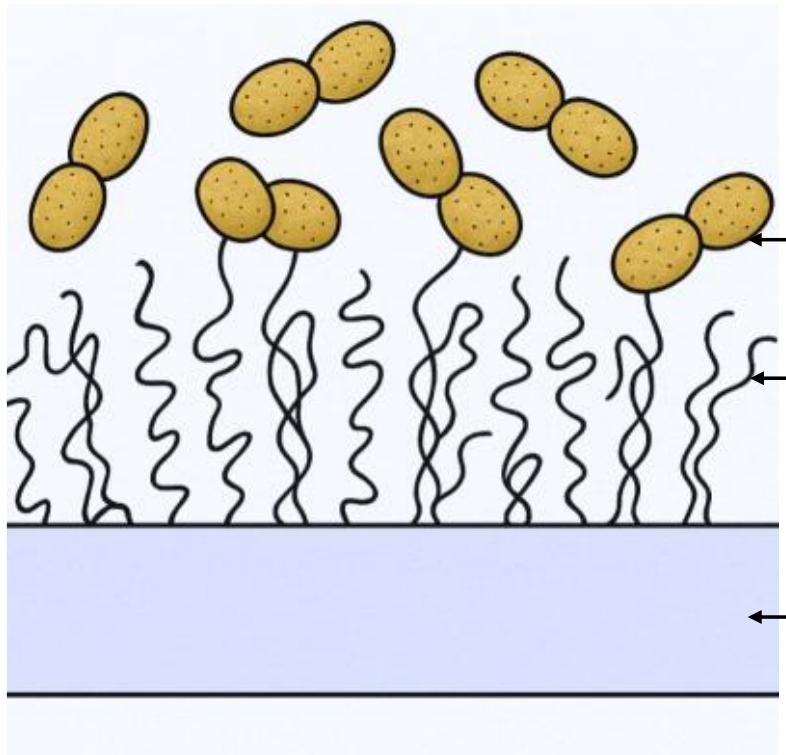

Figure S1: Schematic representation of a biomaterial coated with proteins and a possible bacterial adhesion.

3. Recommended Literature:

[1] H.Y. Erbil, *Surface Chemistry of Solid and Liquid Interfaces*, Blackwell Publishing (2006).

[2] M.E. Schrader, *Young-Dupre Revisited*, Langmuir, 11, 3585-3589.

[3] Li, X., Wang, Z., & Wei, G. (2023). *Advances in surface modification of polycaprolactone for tissue engineering applications*. Journal of Biomedical Materials Research Part A, 111(5), 899–915. https://doi.org/10.1002/jbm.a.37428

[4] Nandakumar, A., et al. (2022). *Protein adsorption and its effects on cell adhesion on polymeric biomaterial surfaces: A review*. Biomaterials Science, 10(7), 1850–1867. https://doi.org/10.1039/d1bm01589a

[5] Kim, H. Y., & Park, S. H. (2024). *Biomolecule-functionalized polycaprolactone scaffolds for enhanced tissue regeneration and antibacterial activity*. ACS Applied Materials & Interfaces, 16(10), 13214–13226. https://doi.org/10.1021/acsami.3c18274

4. Laboratory safety procedures:

Although this lab work presents minimal risk, it is critical to follow standard safety protocols and handle all reagents with care. Since we are dealing with biological material, specifically bacteria, it is essential to work close to the flame. In addition, the safety data sheets of the reagents should be consulted before starting the experiment. No solution is subject to cause irritation.

5. Reagents:

Table S1: Chemicals used in the laboratory class

Material	Grade / Molecular weight	Supplier
Human Serum Albumin (HSA)	≥97%; 66.5 kDa	Sigma-Aldrich (Germany)
Polycaprolactone (PCL)	80 kDa	Sigma-Aldrich (Germany)
Phosphate Buffered Saline (PBS)	pH 7.4, 10 mM phosphate buffer, 2.7 mM potassium chloride, and 137 mM sodium chloride)	VWR International (USA)
Tryptic Soy Broth (TSB)	Suitable for microbiological culture	Difco (USA)
Agar	Suitable for bacteriological use	Liofilchem (Italy)
Human plasma fibronectin (FN)	0.1% solution	Sigma-Aldrich (Germany)
Milli-Q Water	n.a.	n.a.

6. Planning of the sessions:

Table S2: Planning of laboratory sessions.

**Session number**	**General Topic**	**Tasks**
1	Sample preparation and protein adsorption	Sample washing steps.<br>Adsorption of fibronectin and human serum albumin
2	Bacterial adhesion assay	Immersion in a bacterial solution<br>Sonication/serial dilution and plate
3	Contact angle	Measurmert of the surface tention of the PBS by the pendent drop method<br>Measurment of the contact angles by the sessil drop method
4	Bacterial adhesion assay quantification	Colony counting, results analysis

7. Experimental Procedure:

Part 1 – Preparation of protein-coated polycaprolactone samples

1. Cut 9 PCL samples previously prepared into 3 mm cubes.

Note: Each condition was performed in triplicate.

2. Wash 9 samples twice with PBS.

3. Prepare 5 mL of the following solutions: human serum albumin (HSA) 200 µg/mL; fibronectin (FN 20 µg/mL) in PBS.

2. Do a pre-immersion of the PCL samples in the different solutions (Figure S2):
   a. PBS (0.01 M) (3 PCL samples)
   b. Human serum albumin (HSA) (200 µg/mL) (3 PCL samples)
   c. Fibronectin (FN 20 µg/mL) (3 PCL samples)

3. Incubate for 60 min at 37°C.

4. Rinse the wells 3x with PBS (leave the samples in PBS from the last rinse).

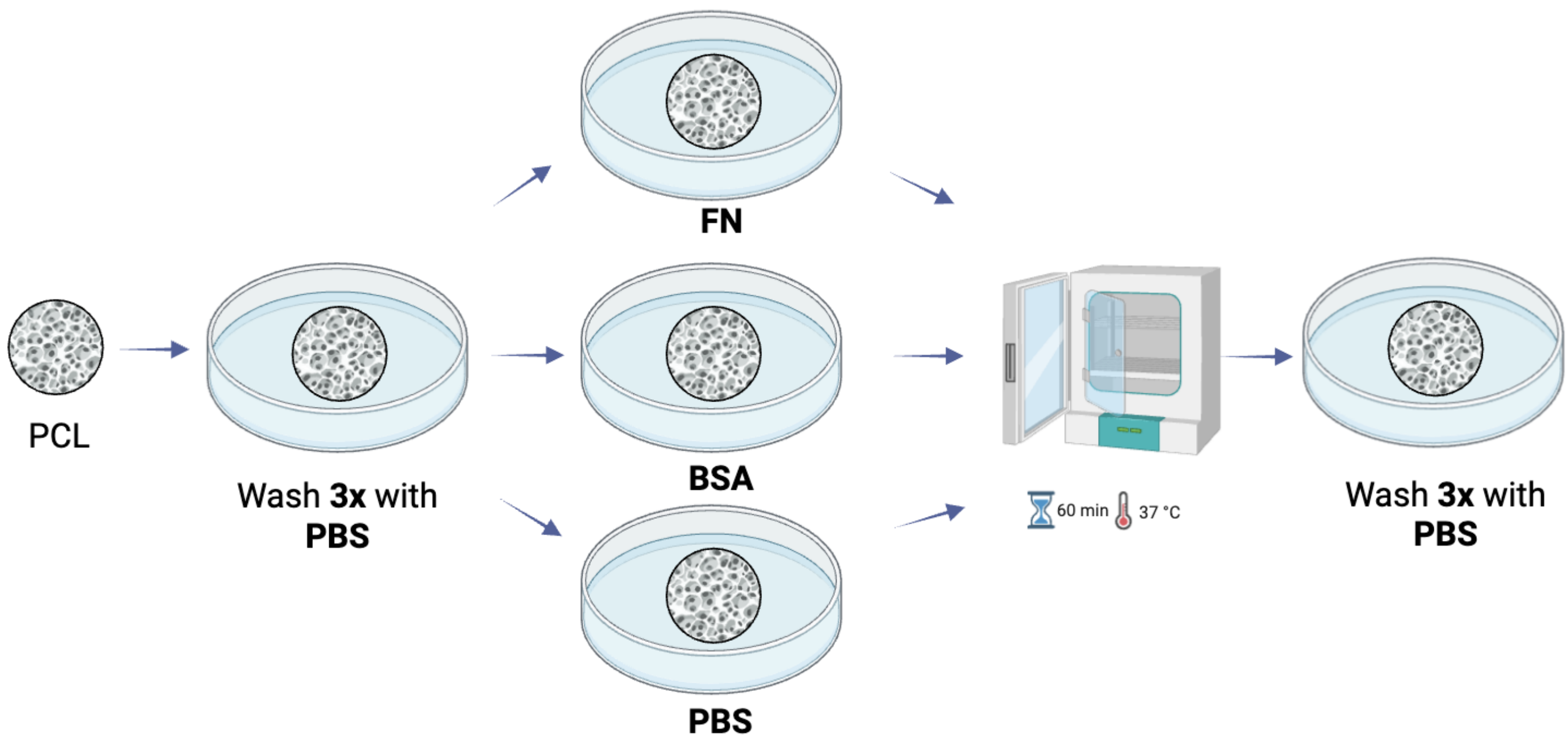

Figure S2: Schematic representation of the PCL samples preparation.

<u>Note:</u> For the evaluation of the protein adsorption to the biomaterial surface, for pedagogical purposes, samples of high-density polyethylene (HDPE) were used for contact angle measurements.

5. Do a pre-immersion of the HDPE samples in the different solutions (Figure S3):
   a. PBS (0.01 M) (3 HDPE samples)
   b. Human serum albumin (HSA) (200 µg/mL) (3 HDPE samples)
   c. Fibronectin (FN 20 µg/mL) (3 HDPE samples)
6. Incubate for 60 min at 37°C.

   4. Rinse the wells 3x with PBS (leave the samples in PBS from the last rinse).

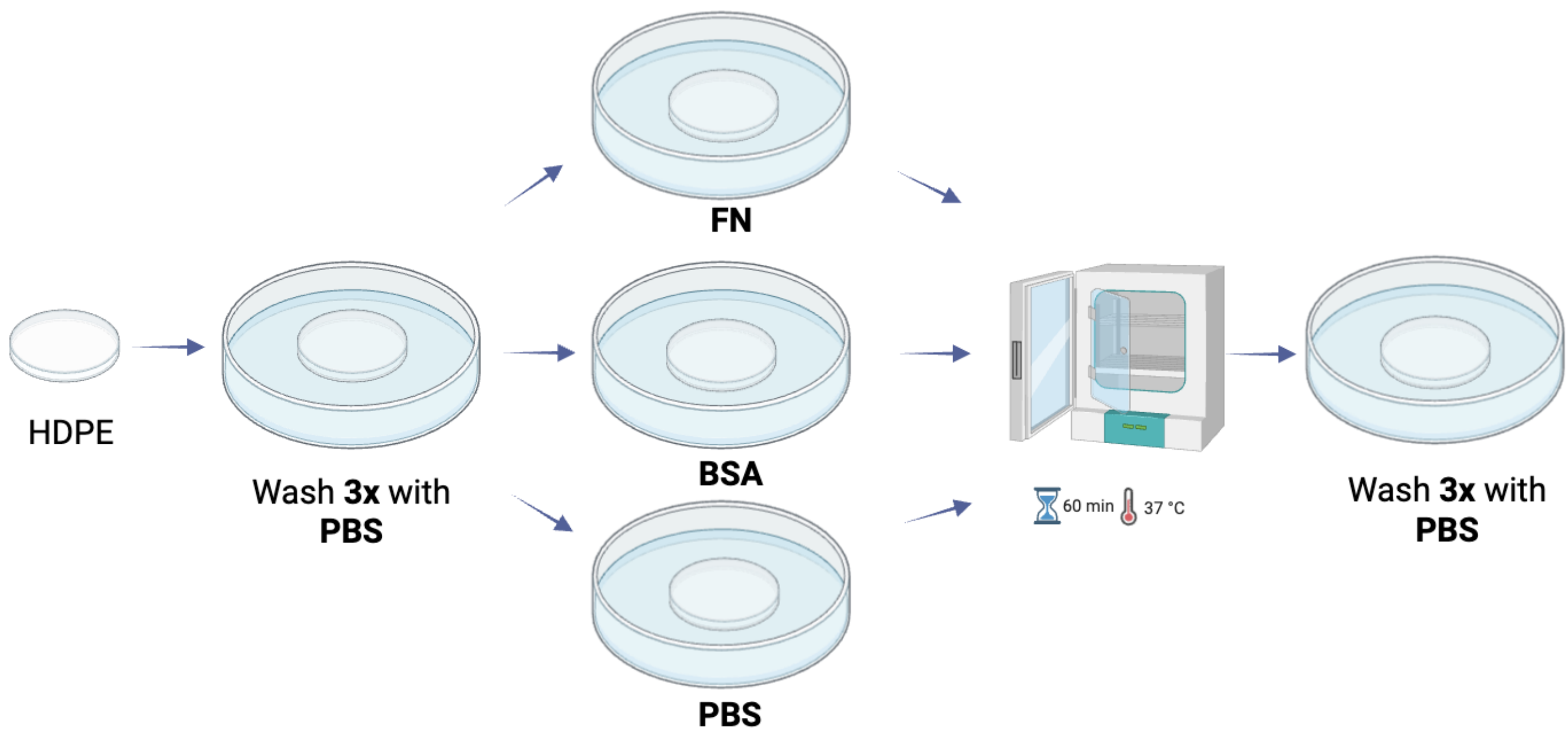

<u>Figure S3</u>: Schematic representation of the HDPE samples preparation.

<u>Part 2 – Bacterial adhesion</u>

1. Prepare a bacterial suspension of 1.0×10^8 colony-forming units (CFU)/mL. For that, in the spectrophotometer, at a wavelength of 640 nm, adjust the absorbance between 0.12 and 0.15 with sterile PBS using the initial *Staphylococcus Epidermidis (S. Epidermidis)* suspension provided by the professor.

2. Incubate the samples prepared in Part 1 with 100 µL 10^8 CFU/mL bacterial solution prepared in step 4 (Figure S4).

3. Incubate 1 h at 37 °C in a horizontal shaker at 70 rpm.

4. Rinse the samples 3x with 100 µL of PBS.

5. Place the samples in a clean well.

6. Ultrasound the samples in 100 µL PBS for 30 seconds.

7. Remove the PBS, perform five serial dilutions, and plate 10 µL of each dilution in TSA using the micro-drop method.

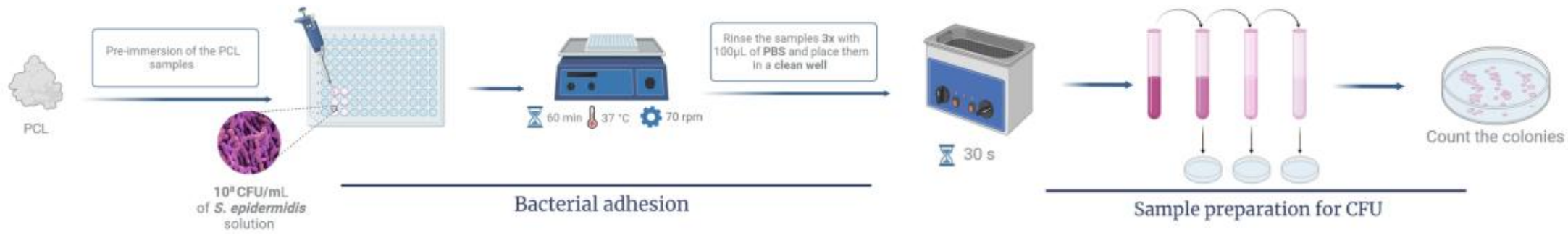

Figure S3: Schematic representation of the bacteria treatment for further CFU counting.

Part 3 – Counting of the bacteria adhered to the polycaprolactone samples

1. After the incubation, count the CFUs for each sample and dilution. Countings under 10 and above 100 are not considered statistically significant.

Part 4 – Evaluation of the contact angles between the biological simulated buffer and the biomaterials

1. Fill the syringe needle with PBS buffer.
2. Dispense a PBS drop and measure the surface tension at room temperature by the hanging drop method using an optical tensiometer. Repeat the experiment until 3 concordant measurements are reached (Figure S5).
3. Measure the contact angles of the PBS on HDPE uncoated with proteins. Repeat the experiment until 3 concordant measurements are reached.

4. Repeat the procedure for surfaces previously coated with HSA and fibronectin.

<u>Note:</u> *double-check if the volumes used in each drop are similar, in order to allow the comparisons of the results.*

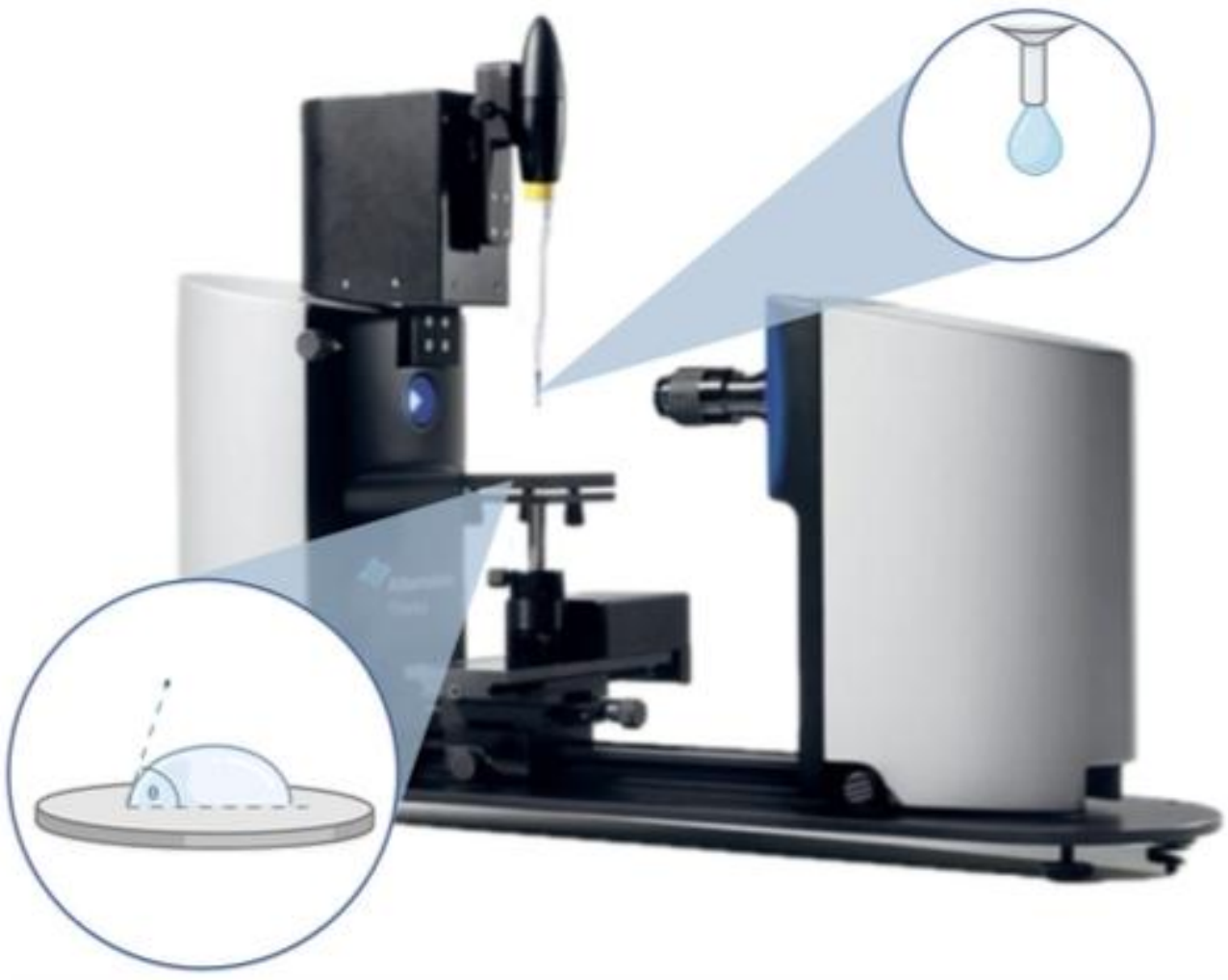

<u>Figure S5</u>: Schematic representation of the measurement of the surface tension by the hanging drop method and the contact angles.

8. Results Discussion

Experimental Results Discussion

Student: ____ Date: ________________

Group I (1 value/question)

Indicate whether the following statement is true or false:

1. Cell adhesion is desirable for the success of medical implants and biomaterials. ___
2. Infections associated with biomaterials cause disabling diseases. ___
3. The assessment of bacterial adhesion to biomaterials in the lab sections was carried out by qualitative methods. ___
4. The use of albumin is related to its great abundance. ___
5. The initial concentration of bacteria (10^6CFU/ml) was adjusted by measuring absorbance at 640 nm. ___
6. The purpose of using ultrasound was the disruption of the bacterial cell membrane. ___
7. In the case of counting 15 colonies in the sample with a dilution factor of 10^2, we are facing an adhesion of bacteria of $1.5x10^3$ CFU/mL. ___
8. Considering the bacterial culture shown in the image, it was found that there was an error in the sample processing. To avoid this error, a larger sample volume should have been used. ___

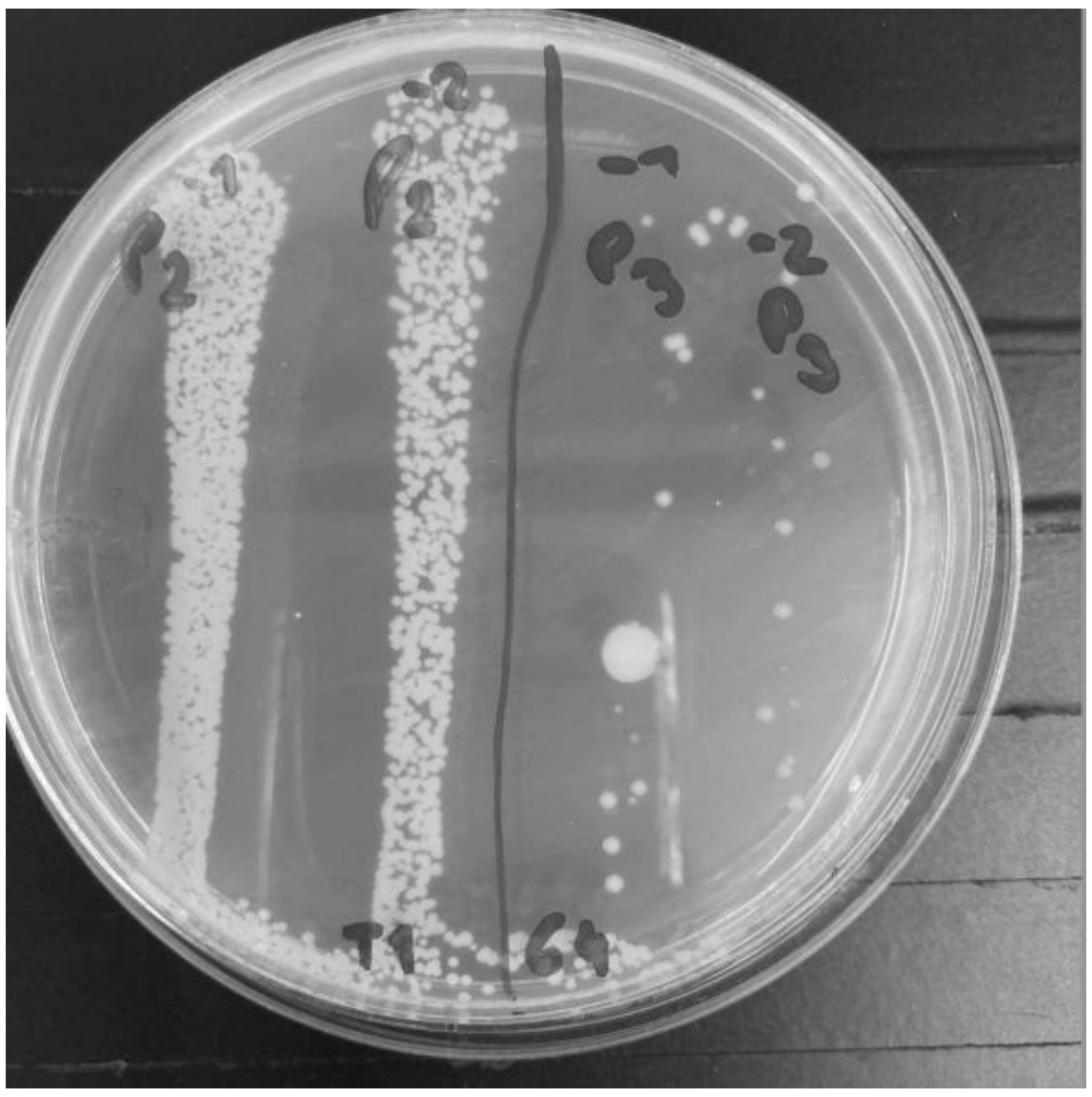

9. The work of cohesion is dependent on the surface tension of the liquid, as well as the surface under analysis. ___

10. The contact angle of a drop of water on different surfaces (A, B, C, D) was analyzed. The surfaces on the left did not have any type of coating. However, the surfaces on the right were previously coated with a protein (the same protein was used for all coatings). According to the contact angle formed, it can be stated that all surfaces became more hydrophobic when coated with the protein. ___

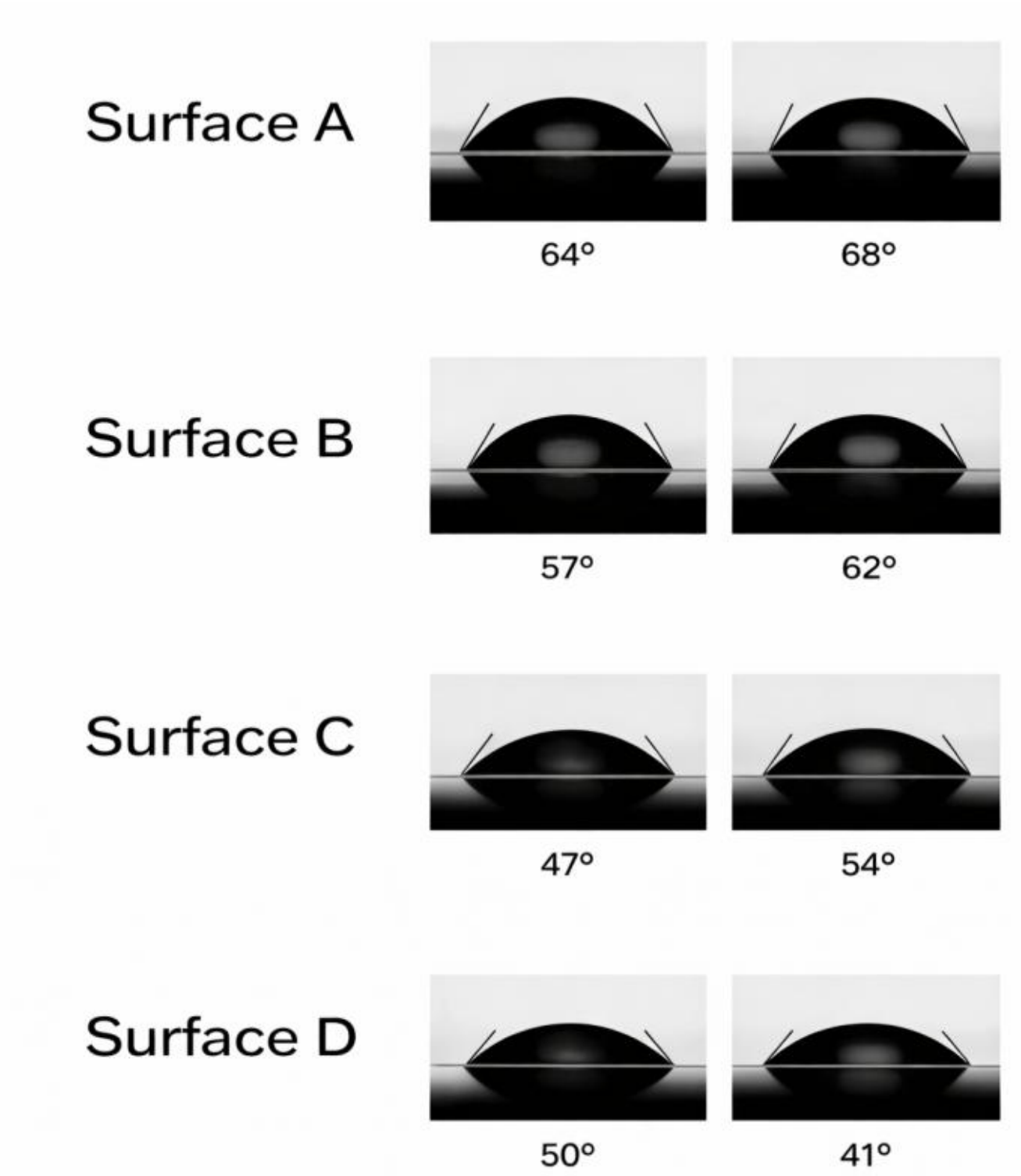

Group II (2 values/question)

1. To what extent would using water instead of PBS in the washing solutions alter the results?

2. What is the use of measuring the optical density of the bacterial solution?

3. Which bacteria was used in the assessment of adherence? What is the reason for choosing this bacterium?

4. As the population of bacteria in a solution is usually very large, it is common for small samples to be used to count. In most cases, the quantification of the bacterial population is done taking into account the number of cells present in 1 ml of liquid medium. Thus, after 4 dilutions were made, 10x, 100x, 1000x, and 1000x, and subsequent cultivation of 20 uL of the solution in petri dishes with culture media, it is possible to determine the number of CFU for each dilution. Based on the counts presented in the following table indicate the concentration of bacteria in the initial solution in CFU/ml.

Fator diluição / dilution factor	10x	100x	1000x	10000x
CFU	> 100	> 100	24	1
	> 100	> 100	13	0
	> 100	98	8	0

5. The contact angle between a liquid with a surface tension of 55 mN/m and a solid surface was determined. The average contact angle obtained was 42°. Taking into account the angle obtained, calculate the adhesion work.

9. Answers to questions

Group I (1 value/question)

Indicate whether the following statement is true or false:

1. **Cell adhesion is desirable for the success of medical implants and biomaterials.** *False*
2. **Infections associated with biomaterials cause disabling diseases.** *True*
3. **The assessment of bacterial adhesion to biomaterials in the lab sections was carried out by qualitative methods.** *False*
4. **The use of albumin is related to its great abundance.** *True*
5. **The initial concentration of bacteria (10^6CFU/ml) was adjusted by measuring absorbance at 640 nm.** *False*
6. **The purpose of using ultrasound was the disrupt the bacterial cell membrane.** *False*
7. **In the case of counting 15 colonies in the sample with a dilution factor of 10^2, we are facing an adhesion of bacteria of $1.5x10^3$ CFU/mL.** *False*
8. **Considering the bacterial culture shown in the image, it was found that there was an error in the sample processing. To avoid this error, a larger sample volume should have been used.** *False*

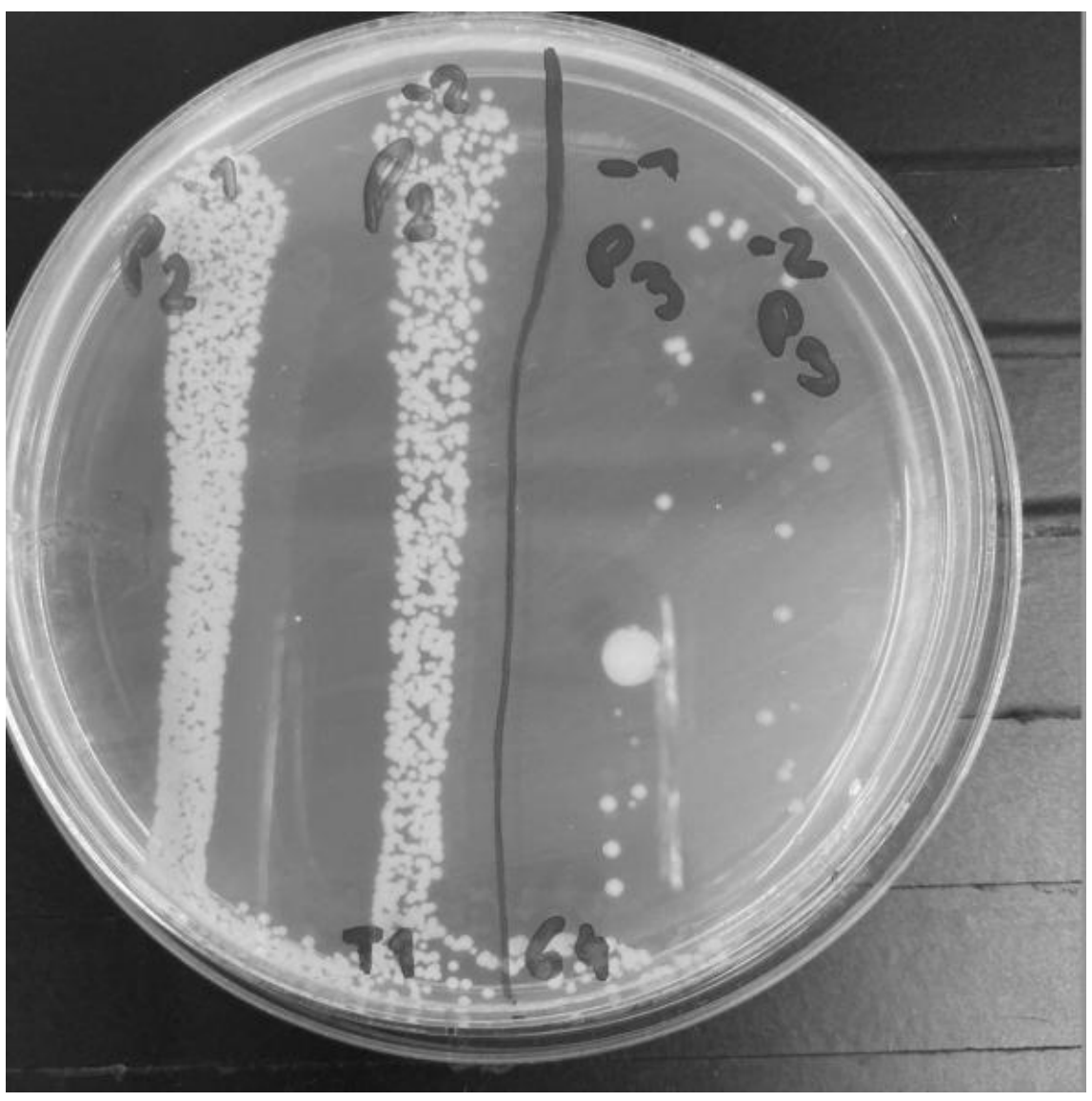

9. **The work of cohesion is dependent on the surface tension of the liquid, as well as the surface under analysis.** *False*
10. **The contact angle of a drop of water on different surfaces (A, B, C, D) was analyzed. The surfaces on the left did not have any type of coating. However, the surfaces on the right were previously coated with a protein (the same protein was used for all coatings). According to the contact angle formed, it can be stated that all surfaces became more hydrophobic when coated with the protein.** *False*

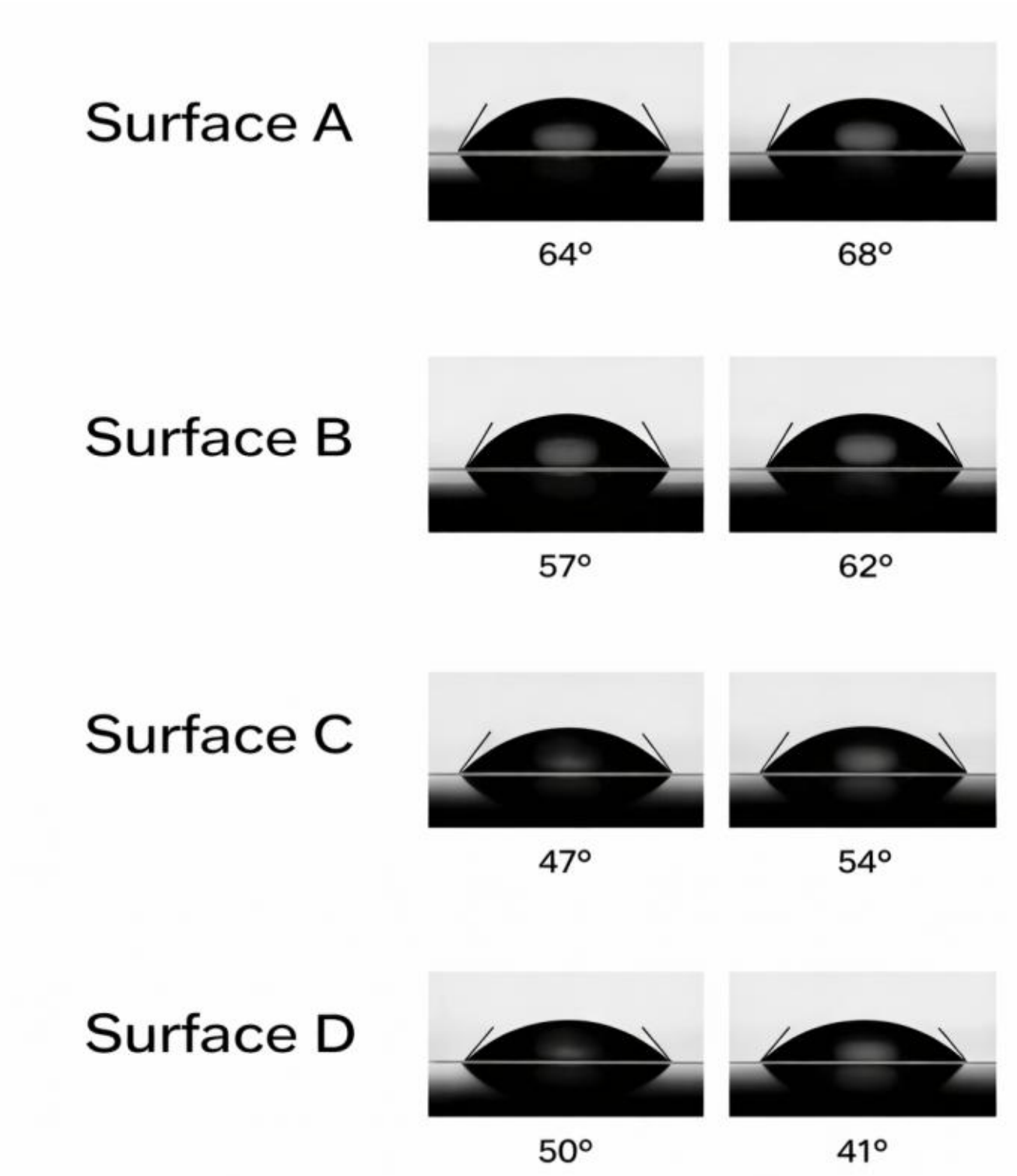

Group II (2 values/question)

1. **To what extent would using water instead of PBS in the washing solutions alter the results?**

The use of water not only fails to properly simulate the physiological environment but also has inadequate osmotic and pH values. PBS is a solution that more closely resembles the physiological values of body fluids.

2. **What is the use of measuring the optical density of the bacterial solution?**

The optical density measurement was used to prepare a bacterial suspension of 1.0 × 10^8 colony-forming units (CFU)/mL, following the McFarland standard. For that, in the spectrophotometer, at a wavelength of 640 nm, absorbance was adjusted to between 0.12 and 0.15 with sterile PBS.

3. **Which bacteria was used in the assessment of adherence? What is the reason for choosing this bacterium?**

The chosen bacterium was S. epidermidis. It was selected because it is considered a commensal skin bacterium, therefore not presenting risks for immunocompetent individuals. However, it is a well-known biofilm producer and is associated with medical device-related infections.

4. **As the population of bacteria in a solution is usually very large, it is common for small samples to be used to count. In most cases, the quantification of the bacterial population is done taking into account the number of cells present in 1 ml of liquid medium. Thus, after 4 dilutions were made, 10x, 100x, 1000x, and 1000x, and subsequent cultivation of 20 uL of the solution in petri dishes with culture media, it is possible to determine the number of CFU for each dilution. Based on the counts presented in the following table indicate the concentration of bacteria in the initial solution in CFU/ml.**

Dilution factor	10x	100x	1000x	10000x
CFU	> 100	> 100	24	1
	> 100	> 100	13	0
	> 100	98	8	0

To calculate the amount of CFU/ml, first, we need to select the dilution that presents between 10 and 100 CFU.

Number of CFUs taking into account the dilution

Sample 1: 24 x 1 000 = 24 000 CFUs

Sample 2: 13 x 1 000 = 13 000 CFUs

Sample 3: 98 x 100 = 9 800 CFUs

Average number of CFU = (24 000 + 13 000 + 9 800) / 3 = 15 600 CFUs

Amount of CFU per mL:

20 uL -------- 15 600 CFU

1000 uL ----- x

$x = 7{,}8 \times 10^{5}$ *CFU / mL*

5. The contact angle between a liquid with a surface tension of 55 mN/m and a solid surface was determined. The average contact angle obtained was 42°. Taking into account the angle obtained, calculate the adhesion work.

$\gamma = 55 \times 10^{-3}$ *N/m*

Adhesion work $= \gamma \times (1 + \cos \theta) = 0.096\ J/m^{2}$

10. Students' Grades

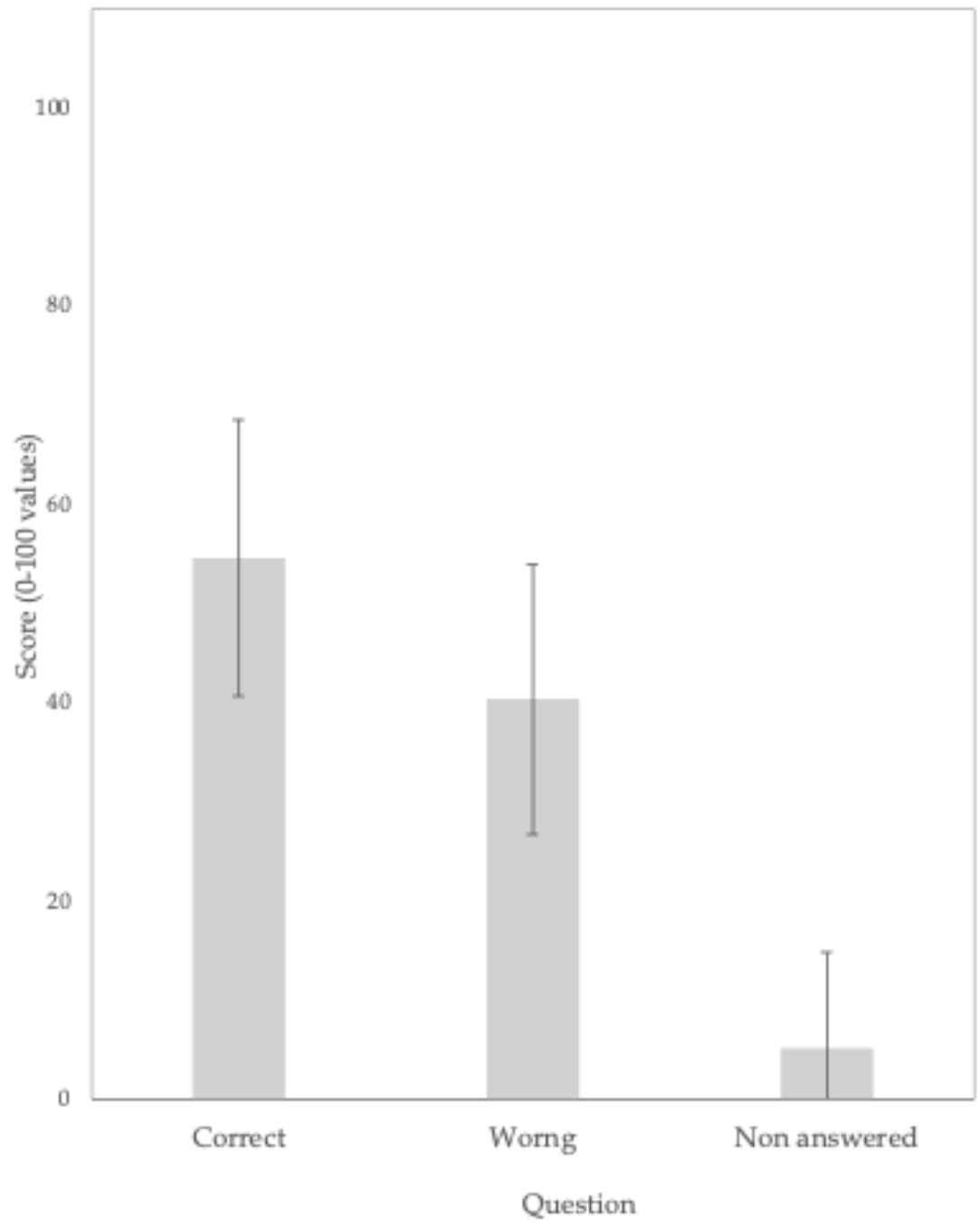

Figure S6: Students' scores in Group I before attending the laboratory practical class (n=57).

Mapping between learning objectives and assessment:

1) Understanding of protein adsorption effects. Questions 1-8 from group I and Questions 2, 4 from group II
2) Calculation of adhesion and cohesion work. Question 5 from group II
3) Evaluation of contact angle interpretation and wettability concepts. Questions 9-10 from group I
4) Integration of physicochemical properties with bacterial adhesion results. Question 1, 3 from group II

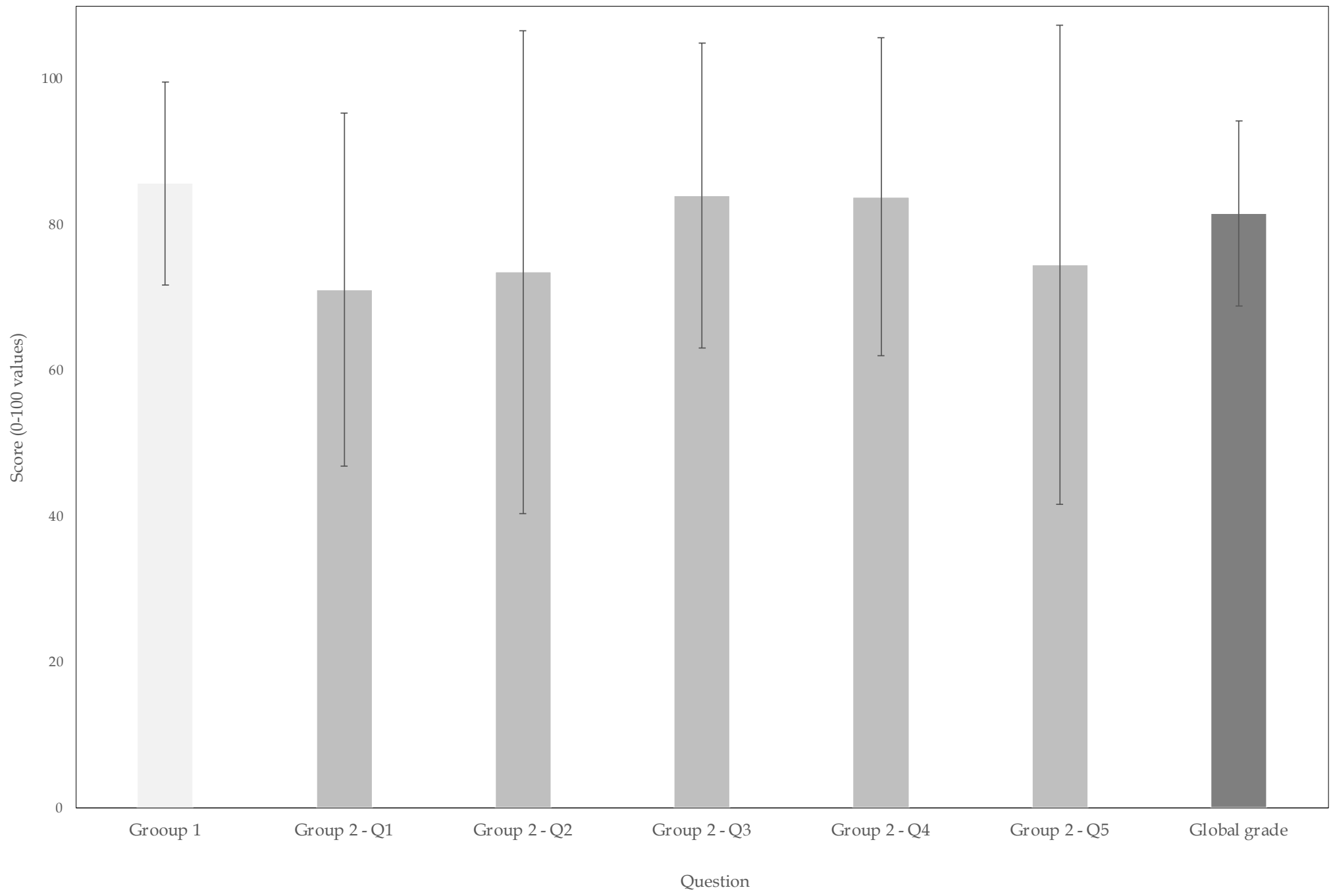

Figure S7: Students' scores by question and group, and the respective final score (n=57).

11. Notes for the instructors

Duration of the experiment: 4 sessions of 2 hours each

Table S3: Duration of each task in each laboratory session.

	Step	Duration (min)
Part I	Cut and wash the PCL samples	20
	Prapare the solutions of FN and HSA	20
	Imerse the PCL and HDPE samples in the different solutions	10
	Incubate the PCL and HDPE samples in the different solutions	65
	Rinse the samples	5
Part II	Bacterial suspention preparation	20
	Incubation of the samples prepared in part I in the bacterial suspension	65
	Rinse the sample	5
	Ultrasound the samples	10
	Plating the samples	20

Part III	Count the CFUs	100
	Discuss about the obtained results	20
Part IV	Measurement of PBS surface tension	30
	Measurement of the contact angles	60
	Calculation of the adhesion and cohesion works	30

Groups: Each group can be composed of up to 5 students

Solutions/materials to be prepared before the class:

a) PCL scaffolds

PCL scaffolds were prepared previously by dissolving 1g of PCL in 50 mL of dioxane (2% w/v) under medium agitation for 1h at room temperature. 15 mL were deposited in a glass petri dish and let it frozen for 24h at -80°C. Finlay, the sample was subjected to lyophilization for 24h to remove all the dioxane.

b) TSB

TSB was prepared according to the manufacturer's instructions. 30 g of powder was suspended in 1 L of ultrapure water and mixed until complete solubilization. Then, the mixture was sterilized at 121°C for 15 minutes, allowed to cool for further use.

c) TSA

To prepare TSA, 30 g of TSB powder and 10 g of Agar were suspended in 1 L of ultrapure water and mixed until complete solubilization. Then, the mixture was sterilized at 121°C for 15 minutes, allowed to cool to 45–50°C and distribute in disposable petri plates.

d) PBS

PBS was produced by dissolving one tablet in 100 mL. Then the solution needs to be filtered with a 0.2 µm filter for further autoclaving.

e) *S. epidermidis* suspension

S. epidermidis reference strain RP62A (ATCC 35984), previously stored at −70 °C in a cryopreservation solution composed of 70% TBS and 30 % glycerol (diluted 1:1 with water), was used. For cultivation, *S. epidermidis* strain was cultured on TSB at 37 °C for 24 hours. After incubation, 50 µL of the bacterial culture was transferred to a tube containing 30 mL of fresh TSB and incubated again at 37°C with gentle shaking for 18 hours to achieve a stationary phase. The bacterial cells were then collected by centrifugation for 5 minutes at a force of 10 N. The harvested cells were washed twice and resuspended in a saline solution (0.9% NaCl in distilled water) at a final concentration of 1.0×10^8 CFU/mL, following the McFarland standard (BioMerieux, France).

Material:

Lab Material and Equipment	Quantity per group
Petri plates	15
100-1000 µL micropipette tip	4
100-1000 µL micropipette	1
Incubator	1*
Chronometer	1
Spectrometer UV-vis	1*
Orbital incubator	1*
Ultrasound bath	1*
Disposable cuvette	1
Optical Tensiometer	1*

* One equipment per class.

Waste Disposal

Disposal of materials used in tests with biological components, such as bacteria, must follow biosafety protocols to avoid contamination and health risks.

a) Contaminated materials (petri dishes, tips, tubes, swabs, etc.) must be autoclaved at 121°C for 15-20 minutes before disposal.

b) Personal protective equipment (PPE) and single-use materials such as gloves, countertop papers, etc. should be placed in biohazard bags and autoclaved before disposal.
